# Supplementary material for: Effects of primary health care and socioeconomic aspects on the dispersion of COVID-19 in the Brazilian Northeast: Ecological study of the first pandemic wave
Source: PLoS One. 2024 Mar 27;19(3):e0296837. doi: 10.1371/journal.pone.0296837 (PMC10971773; doi:10.1371/journal.pone.0296837)
Supplement: Attachment — Submitted filename: CARTA RESPOST1.pdf [file pone.0296837.s002.pdf]

ANSWER LETTER

Dear editor, greetings!

I come, through this, to answer the questions sent via the platform of the manuscript “ *Effects of Primary Health Care and socioeconomic Aspects on the Dispersion of COVID-19 in the Brazilian Northeast : Ecological study of the First Pandemic wave* ”

In response to Pending 01 “Rebuttal letter describing response to original reviewers’ comments”

- The manuscript was returned due to the lack of a cover letter, it was constructed and sent together with the manuscript, there were no changes or comments from the reviewers.

In response to Pending 02 “Version Track Changes ”

- There were no modifications to the original manuscript, the text was returned due to the need for a cover letter and there were no comments for the reformulation of the text.
